# Up-Regulation of Claudin-6 in the Distal Lung Impacts Secondhand Smoke-Induced Inflammation

**DOI:** 10.3390/ijerph13101018

**Published:** 2016-10-17

**Authors:** Joshua B. Lewis, Dallin C. Milner, Adam L. Lewis, Todd M. Dunaway, Kaleb M. Egbert, Scott C. Albright, Brigham J. Merrell, Troy D. Monson, Dallin S. Broberg, Jason R. Gassman, Daniel B. Thomas, Juan A. Arroyo, Paul R. Reynolds

**Affiliations:** Lung and Placenta Research Laboratory, Physiology and Developmental Biology, Brigham Young University, Provo, UT 84602, USA; jblewis1101@gmail.com (J.B.L.); dallinmilner@gmail.com (D.C.M.); adambyulewis@gmail.com (A.L.L.); toddmdunaway@gmail.com (T.M.D.); kaleb_egbert@hotmail.com (K.M.E.); scottcalbright@gmail.com (S.C.A.); brighammerrell@gmail.com (B.J.M.); tmon92@gmail.com (T.D.M.); dallin.broberg@gmail.com (D.S.B.); jgassman15@gmail.com (J.R.G.); dbthomas27@gmail.com (D.B.T.); jarroyo@byu.edu (J.A.A.)

**Keywords:** Claudin-6, lung, tobacco, transgenic

## Abstract

It has long been understood that increased epithelial permeability contributes to inflammation observed in many respiratory diseases. Recently, evidence has revealed that environmental exposure to noxious material such as cigarette smoke reduces tight junction barrier integrity, thus enhancing inflammatory conditions. Claudin-6 (Cldn6) is a tetraspanin transmembrane protein found within the tight junctional complex and is implicated in maintaining lung epithelial barriers. To test the hypothesis that increased Cldn6 ameliorates inflammation at the respiratory barrier, we utilized the Tet-On inducible transgenic system to conditionally over-express Clnd6 in the distal lung. Cldn6 transgenic (TG) and control mice were continuously provided doxycycline from postnatal day (PN) 30 until euthanasia date at PN90. A subset of Cldn6 TG and control mice were also subjected to daily secondhand tobacco smoke (SHS) via a nose only inhalation system from PN30-90 and compared to room air (RA) controls. Animals were euthanized on PN90 and lungs were harvested for histological and molecular characterization. Bronchoalveolar lavage fluid (BALF) was procured for the assessment of inflammatory cells and molecules. Quantitative RT-PCR and immunoblotting revealed increased Cldn6 expression in TG vs. control animals and SHS decreased Cldn6 expression regardless of genetic up-regulation. Histological evaluations revealed no adverse pulmonary remodeling via Hematoxylin and Eosin (H&E) staining or any qualitative alterations in the abundance of type II pneumocytes or proximal non-ciliated epithelial cells via staining for cell specific propeptide of Surfactant Protein-C (proSP-C) or Club Cell Secretory Protein (CCSP), respectively. Immunoblotting and qRT-PCR confirmed the differential expression of Cldn6 and the pro-inflammatory cytokines TNF-α and IL-1β. As a general theme, inflammation induced by SHS exposure was influenced by the availability of Cldn6. These data reveal captivating information suggesting a role for Cldn6 in lungs exposed to tobacco smoke. Further research is critically necessary in order to fully explain roles for tight junctional components such as Cldn6 and other related molecules in lungs coping with exposure.

## 1. Introduction

In recent decades, research surrounding tight junctional proteins has increased and novel insights have revealed much about the function of tight junctions in health and disease. Initially, tight junctions were believed to be simple paracellular seals; however, it is now understood that tight junctions additionally act as key mediators in regulating cell polarity [1] and in moderating cellular proliferation and differentiation [2]. Tight junctions are responsible for maintaining the epithelial permeability barrier and thus they also contribute to the innate immune system. In regards to the lung, tight junctions function in the respiratory defense system by protecting tissues from harmful exogenous materials such as allergens, air pollution, and cigarette smoke constituents [3,4]. In addition, tight junctions help regulate the availability of healthy endogenous material and fluids across epithelial barriers [5]. The loss of such critical functions have been implicated in the pathogenesis of many debilitating conditions such as chronic obstructive pulmonary disease (COPD) [6,7], Emphysema [8,9], Pulmonary Fibrosis [10,11], and acute respiratory distress syndrome (ARDS) [12,13,14]. Together, these conditions represent an enormous burden on the healthcare system. As one example, COPD is currently the fourth leading cause of death worldwide and is predicted to become the third leading cause of death by 2030 [15]. The economic burden of COPD alone is in the range of $18 billion annually [16], and is only expected to increase as death rates similarly rise [17]. While the mechanisms surrounding the development of COPD are incompletely characterized, it is known that cigarette smoke is a major risk factor in the development of the condition [18]. Thus, as current COPD trends are estimated to rise in the coming years, more research surrounding the possible contributions of tight junctions and the effects on maintaining epithelial barrier integrity may provide novel insights into therapeutic avenues that may aid in combating the disease.

Claudin (Cldn) proteins are critical participants that aid in the establishment of barriers that contain tight junctions [19]. Claudins are tetraspanin transmembrane proteins that interact with structural proteins to form the barrier that impacts the selective permeability observed between epithelial cells [20,21]. Cldn-3 and Cldn-18 [11,22] are family members abundantly expressed in the lung; however, additional Cldn proteins have been implicated in lung development and in the maintenance of normal lung physiology [20,23,24,25].

Cldn6 is one such member of this family of proteins whose role in lung physiology has only recently begun to be elucidated. The normal expression pattern of Cldn6 peaks during early embryogenesis (E14.5 in mice) and gradually decreases to lower yet detectible levels at birth [26] and through adulthood. TTF-1, Gata-6, and FoxA2, are all transcription factors that are known to regulate critical gene programs that control pulmonary epithelial cell differentiation during lung morphogenesis [27,28]. Interestingly, these same transcription factors have been shown to regulate Cldn6 expression during pulmonary development [26]. Precise transcriptional control of Cldn6 is implicated due to the recent discovery that the overexpression of Cldn6 results in a notable delay of lung morphogenesis [29]. Cldn6 may also be implicated in the development of diverse cancers [30,31,32]. In relation to lung cancer, current evidence suggests a role for Cldn6; however, reports disagree as to whether the presence of Cldn6 confers improved or diminished prognoses [25,33]. While incompletely understood, these data suggest that Cldn6 may mediate critical functions during development and normal adult life. In summary, the evidence supports the idea that deviations from its normal adult pattern of expression may have consequences related to tight junctional effects in compromised adult lungs, however whether those consequences are advantageous or deleterious remain to be elucidated.

The aim of this investigation was to determine the effects of overexpressed Cldn6 in the adult lung, and more specifically determine its potential to protect against inflammation resulting from the exposure to destructive noxious material associated with cigarette smoke.

## 2. Materials and Methods

### 2.1. Mice

Two transgenic lines of mice were developed from a C57Bl/6 background (Jackson Laboratories, Bar Harbor, ME, USA) and used to create conditional doxycycline (dox)-inducible mice that overexpress Cldn6 in the distal lung. Briefly, one line of mice was generated in our laboratory that harbored a single transgene that contained the Cldn6 cDNA sequence downstream of seven concatomerized Tet-on response elements [34]. These mice were mated to mice of a second transgenic line that express a reverse tetracycline transactivator (rtTA) downstream of the human surfactant protein C (SP-C) promoter [35]. Male double transgenic SPC-rtTA/TetO-Cldn6 mice (Cldn6 TG) were weaned and genotyped at PN20 [36] and dox (625 mg/kg; Harlan Teklad, Madison, WI, USA) was continuously available until euthanasia on PN90. Single or non-transgenic mice were used as controls and were given the same regimen of dox administration. At time of necropsy, *en bloc* lungs were inflation fixed with 4% paraformaldehyde for histology, lavaged for procurement of bronchoalveolar lavage fluid (BALF) [37], or resected prior to the isolation of total protein or RNA [37]. Mice were housed and utilized in accordance with protocols approved by the Institutional Animal Care and Use Committee (IACUC) at Brigham Young University (Protocol number 15-0403).

### 2.2. Secondhand Smoke Exposure

As noted, select mice were exposed to SHS generated from 3R4F research cigarettes from Kentucky Tobacco Research and Development Center, University of Kentucky, via a nose-only exposure system (InExpose System, Scireq, Montreal, QC, Canada) as outlined previously [38]. Briefly, Cldn6 TG and control mice were exposed to SHS generated from 2 research cigarettes during a 10-min period each weekday from PN30 until necropsy on PN90. For comparison purposes, Cldn6 TG and control mice were similarly restrained for the same duration and were exposed to room air (RA). The SHS challenge was determined to be an acceptable level of particulate density concentration according to [39,40], and was tolerated without evidence of toxicity. The specific total particulate density concentration was measured weekly and an average of 132.6 mg total particulate matter per cubic meter in the tower was detected. Furthermore, this nose only model of smoke exposure yielded chronic blood carboxyhemoglobin levels of ~5%, a value similarly observed in human smokers [41].

### 2.3. Histology and Immunohistochemistry

Cldn6 TG and non-transgenic control lungs were fixed in 4% paraformaldehyde, processed, embedded and sectioned at 4 µm thickness [42]. Classic hematoxylin and eosin (H&E) staining was performed to observe general lung morphology. Immunostaining for cell-specific markers followed slide dehydration, deparaffinization, and processing with antigen retrieval by citrate buffer. Antibodies that were used include: anti-Cldn6 goat polyclonal antibody (C-20, 1:100; Santa Cruz Biotechnologies, Santa Cruz, CA, USA) CCSP (CCSP, WRAB-3950, 1:100 Seven Hills Bio Reagents, Cincinnati, OH, USA), and propeptide of Surfactant Protein-C (proSP-C) (proSP-C, WRAB-76694, 1:100 Seven Hills Bio Reagents, Cincinnati, OH, USA).

### 2.4. Immunoblotting

Immunoblotting was performed as previously outlined by our laboratory [43]. Briefly, tissues were homogenized in protein lysis buffer (RIPA, Fisher Scientific, Pittsburg, PA, USA). 20 µg of protein lysates were separated on Mini-PROTEAN^®^ TGX™ Precast gel (Bio-Rad Laboratories, Hercules, CA, USA) and transferred to nitrocellulose membranes. Membranes were blocked and incubated with polyclonal antibodies against Cldn6 (at a dilution of 1:200; Santa Cruz Biotechnology, Santa Cruz, CA, USA), TNF-α (sc-52746, Santa Cruz Biotechnology, Dallas, TX, USA, 1:200) or IL-1β (ab9722, Abcam, Cambridge, MA, USA; dilution 1:200). Secondary (Ig)-horeseradish peroxidase antibodies were added for one hour at room temperature. The membranes were incubated with chemiluminescent substrate (Pierce, Rockford, IL, USA) for 5 min and the emission of light was digitally recorded using a C-DiGit^®^ Blot Scanner (LI-COR, Inc., Lincoln, NE, USA). Immunoblotting was conducted at least twice in triplicate and average band densities were normalized to β-actin densities prior to performing statistical tests.

### 2.5. qRT-PCR

Total RNA was isolated from mouse lungs using an RT-PCR Miniprep Kit (Stratagene, La Jolla, CA, USA). Reverse transcription of RNA in order to obtain cDNA for qRT-PCR and cDNA amplification was performed using Bio Rad iTaq Universal SYBR^®^ Green One-Step Kit (Bio-Rad Laboratories, Hercules, CA, USA). Data analysis was performed using a Bio Rad Single Color Real Time PCR detection system (Bio-Rad Laboratories, Hercules, CA, USA) [2]. The following primers were synthesized by Invitrogen Life Technologies (Grand Island, NY, USA): IL-1β (For-TGT AAT GAA AGA CGG CAC ACC and Rev-TCT TCT TTG GGT ATT GCT TGG), TNF-α (For-TGC CTA TGT CTC AGC CTC TTC and Rev-GAG GCC ATT TGG GAA CTT CT), Cldn6 (For-CAT TAC ATG GCC TGC TAT TC and Rev-CAC ATA ATT CTT GGT GGG ATA TT), and β-actin (For-ACA GGA TGC AGA AGG AGA TTA C and Rev-CAC AGA GTA CTT GCG CTC AGG A).

### 2.6. ELISAs

Molecule-specific ELISA kits that screen for TNF-α or IL-1β (Ray BioTech, Norcross, GA, USA) were used to assess secretion of these two inflammatory cytokines in lung BALF samples. Briefly, lung BALF samples were isolated from control and treatment groups and screened as outlined in the manufacturer’s instructions.

### 2.7. Statistical Analysis

Data were assessed by one- or two-way analysis of variance (ANOVA). When ANOVA indicated significant differences, the Student’s *t*-test was used with the Bonferroni correction for multiple comparisons. The results presented are representatives and *p*-values ≤ 0.05 were considered significant.

## 3. Results

### 3.1. Cldn6 Expression

**Figure 1:**
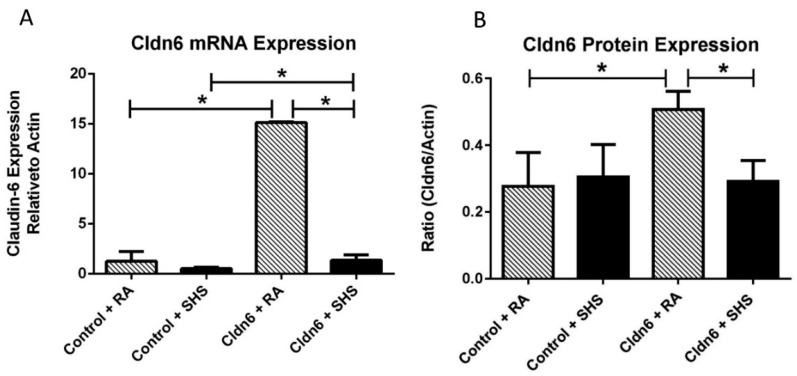
(**A**) Claudin-6 (Cldn6) mRNA expression was significantly decreased in the lungs of control mice following secondhand tobacco smoke (SHS) exposure and up-regulation of Cldn6 in Cldn6 transgenic (TG) mice was inhibited in TG mice exposed to SHS (*n* = 6 per group). The mRNA from each group was normalized to β-actin and representative data are shown with * *p* ≤ 0.05; (**B**) Immunoblotting revealed that Cldn6 protein was unchanged in control mice following SHS exposure and that protein levels were significantly inhibited in Cldn6 TG mice exposed to SHS. Representative blots (*n* = 6 per group) were densitometrically normalized to β-actin and ratios of Cldn6/β-actin are presented with * *p* ≤ 0.05.

Utilizing qRT-PCR, we discovered that Cldn6 mRNA expression was elevated by approximately ~13 fold in Cldn6 TG mice (Figure 1A). To confirm that transcriptional mechanisms corresponded with protein expression, we also analyzed protein levels of Cldn6 and found that although statistically significant, a less robust increase in Cldn6 protein levels occurred (Figure 1B). These data suggest both mRNA and protein are elevated in our genetic models of Cldn6 up-regulation. We next sought to assess Cldn6 expression profiles in the context of SHS exposure. Cldn6 mRNA expression was profoundly diminished in the lungs of Cldn6 TG mice following 60 days of SHS exposure (Figure 1A) and blotting for Cldn6 revealed that SHS similarly decreased protein expression to room air (RA) baselines (Figure 1B). There was no decrease in the expression of Cldn6 protein in controls exposed to SHS.

### 3.2. Lung Morphology

**Figure 2:**
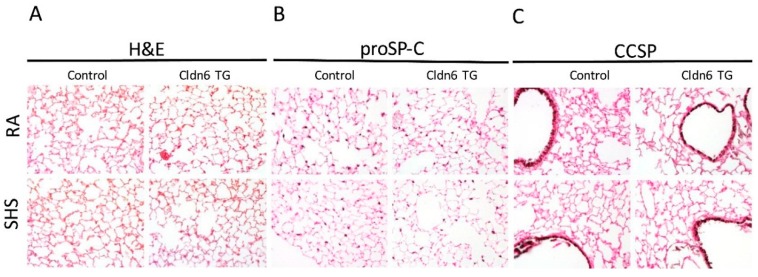
Representative sections of lung tissue were stained with Hematoxylin and Eosin (H&E) (**A**), propeptide of Surfactant Protein-C (proSP-C) (**B**); or Club Cell Secretory Protein (CCSP) (**C**). No observable anatomical disturbances were observed in the respiratory regions or conducting airways (**A**). Furthermore, no qualitative differences were detected in lung sections when alveolar type II cells (**B**) or non-ciliated proximal epithelial cells were counted (**C**). No staining was observed in controls that lacked primary antibody (not shown). Images (200× magnification) are representative of experiments involving four animals from each group.

Histological analysis via H&E staining revealed no evidence of tissue simplification or any discernable changes in general lung morphology (Figure 2A). Immunohistochemical staining was next employed using cell-specific markers in order to assess whether there were any cellular changes in the proximal or distal lung compartments. Staining for the propeptide of Surfactant Protein-C (proSP-C) revealed no alterations in the qualitative abundance of alveolar type II cells in the distal lung (Figure 2B). In fact, there were no discernable differences in the relative abundance of alveolar type II cells in Cldn6 TG or controls regardless of SHS exposure. Immunohistochemical assessment of proximal lung tissue was completed using a marker for Club Cell Secretory Protein (CCSP) elaborated by non-ciliated Club cells in the conducting airways. As was similarly observed relative to proSP-C staining, CCSP immunostaining was consistent in each of the experimental groups and controls, suggesting no significant alterations in proximal cell quantity (Figure 2C).

### 3.3. Pro-Inflammatory Conditions in the Lung

**Figure 3:**
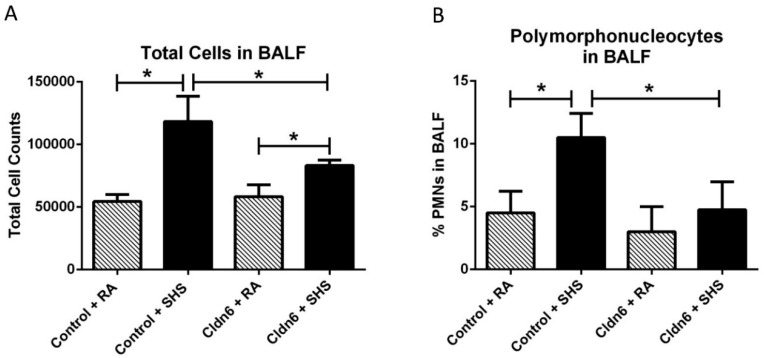
(**A**) Total bronchoalveolar lavage fluid (BALF) cells were significantly increased in control mice + SHS when room air (RA)-exposed controls. There was significantly less total cellularity in BALF from Cldn6 transgenic (TG) mice exposed to SHS when compared to SHS-exposed controls; (**B**) The percentage of polymophonuclear cells (PMNs) was significantly higher in control mice + SHS compared to control mice + RA and PMN quantity was unchanged in Cldn6 TG mice following SHS exposure. Data are representative of experiments involving six mouse lung samples per group and * *p* ≤ 0.05.

**Figure 4:**
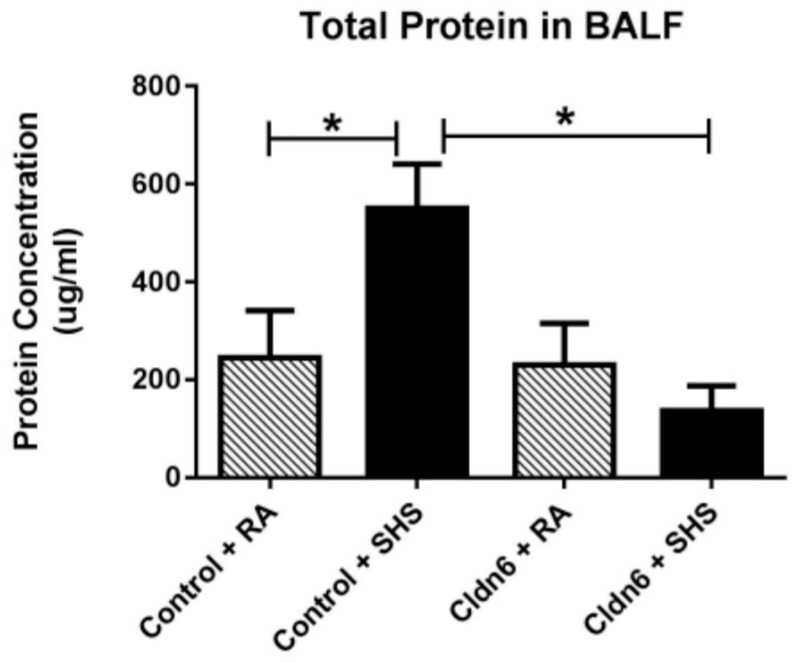
Total BALF protein was assayed using the BCA technique to demonstrate vascular permeability. Protein was significantly elevated in control mice following SHS exposure and there was no change in total BALF protein from Cldn6 TG mice + SHS when compared to RA groups. Data are representative of experiments involving six mouse lung samples per group and * *p* ≤ 0.05.

A battery of assessments aimed at discerning inflammatory profiles was next conducted in order to assess whether Cldn6 expression impacted inflammatory responses to SHS exposure. Quantification of total cells in bronchoalveolar lavage fluid (BALF) resulted in an expected increase in total cells following exposure of control animals to SHS (Figure 3A). While SHS also significantly increased cellular abundance in BALF obtained from Cldn6 TG animals, significantly less total cells were observed in BALF from Cldn6 TG animals exposed to SHS when compared to control SHS-exposed mice (Figure 3A). Differential cell analyses were conducted in order to determine the abundance of polymophonuclear cells (PMNs). These assessments revealed significant inhibition of PMN extravasation into airways of Cldn6 TG mice exposed to SHS compared to SHS-exposed controls (Figure 3B). In addition to the differential elaboration of cells, there was also a significant difference in the quantity of total protein detected in BALF (Figure 4). As a commonly accepted indirect measure of vascular permeability, the assessment of total protein in BALF revealed a marked increase of protein expression in the BALF of control animals exposed to SHS. Interestingly, Cldn6 TG mice experienced no change in BALF protein abundance following SHS exposure (Figure 4).

**Figure 5:**
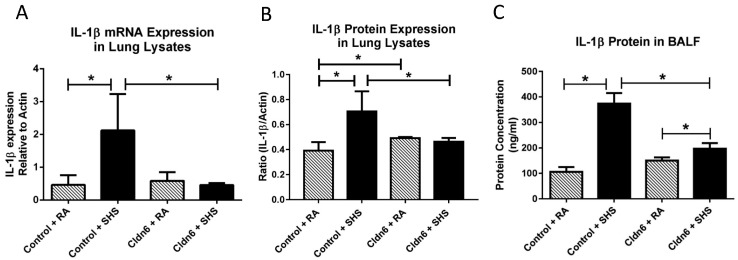
IL-1β mRNA (**A**) and protein (**B**) were assayed in whole lung lysates by qPCR and immunoblotting, respectively. SHS increased IL-1β transcription and translation in control animals, but expression of both message and protein were unchanged in SHS-exposed Cldn6 TG mice; (**C**) ELISAs were used to detect secreted IL-1β in BALF. Despite significant increases in the secretion of IL-1β following SHS exposure, there was significant inhibition of IL-1β in Cldn6 TG mice exposed to SHS when compared to SHS-exposed controls. Data are representative of experiments involving six mouse lung samples per group and * *p* ≤ 0.05.

**Figure 6:**
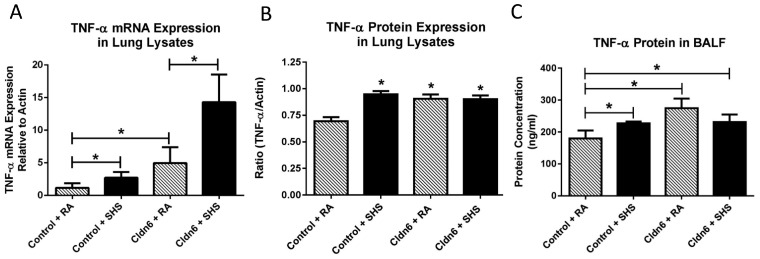
TNF-α mRNA (**A**) and protein (**B**) were assayed in whole lung lysates by qPCR and immunoblotting, respectively. SHS increased TNF-α transcription in both control and Cldn6 TG animals. TNF-α protein in lung lysates was significantly increased in SHS-exposed controls, but no increase was observed in Cldn6 TG mice following SHS exposure; (**C**) ELISAs were used to detect secreted TNF-α in BALF. Secreted TNF-α was increased in control mice following SHS exposure. Despite a slightly higher basal level of secretion by Cldn6 TG mice, SHS did not significantly increase TNF-α secretion. Data are representative of experiments involving six mouse lung samples per group and * *p* ≤ 0.05.

Our attention next focused on the elaboration of pro-inflammatory cytokines. As expected, there was a significant increase in the expression of IL-1β mRNA and protein in whole lung homogenates from control mice after SHS exposure (Figure 5A,B). There was also a significant increase in the elaboration of IL-1β by control mice exposed to SHS into BALF (Figure 5C). Similar assessments also demonstrated that there was no increase in the expression of IL-1β message or protein when total mRNA or protein were screened in lysates from Cldn6 TG mice exposed to SHS (Figure 5A,B). While there was a significant increase in the secretion of IL-1β by Cldn6 TG mice exposed to SHS compared to Cldn6 TG mice exposed to RA, SHS-induced IL-1β secretion was significantly decreased in Cldn6 TG mice compared to controls (Figure 5C). TNF-α is another pro-inflammatory cytokine synthesized and secreted following exposure to tobacco smoke. Our data demonstrate a significant increase in the expression of TNF-α mRNA from lysates obtained from SHS-exposed Cldn6 TG and controls (Figure 6A). Interestingly, TNF-α protein expression in whole lung lysates was elevated, yet not differentially increased in relation to transgenic control programs (Figure 6B). Lastly, despite a higher baseline of secreted TNF-α into BALF by Cldn6 TG mice exposed to RA (Figure 6C), TNF-α secretion by Cldn6 TG mice was not significantly increased following SHS exposure whereas such secretion was markedly increased following SHS exposure of controls (Figure 6C).

## 4. Discussion

The anatomic apposition of the lungs and the environment accounts for the exposure to myriad constituents, each with varying effects on the integrity of pulmonary tissue and the inflammatory status of the individual. Environmental tobacco smoke, or secondhand smoke (SHS), is a key risk factor for pulmonary disease in all studied demographics [44]; however, children are particularly vulnerable due to their lack of control over their living environment. For example, data collected between 2009–2013, from 21 countries in the Global Adult Tobacco Survey (GATS), supported the concept that an estimated 507 million of the 1 billion children residing in the identified countries are exposed to SHS in the home [45]. Without question, identifying molecular mechanisms are necessary so that disease attenuation can be approached in a multifaceted manner. Such an approach should involve cessation programs, direct cellular perturbation, and the exploration into indirect anti-inflammatory modalities. In the current set of experiments, we sought to explore the potential contributions of tight junctions during SHS exposure and hypothesized that more abundant junctions confer measurable anti-inflammatory protection. 

Tobacco smoke exposure has been shown to disrupt pulmonary tight junctions. As one example, work conducted by Schamberger et al. [7] demonstrated that bronchial tight junctional components were reduced following exposure. Olivera et al. further showed that lung epithelial cells undergo multicellular junctional reorganization during smoke-induced permeability abnormalities [46]. These and other data suggest that reversing the targeting of tight junctional components during exposure may prove advantageous in lessening smoke-induced cell stress responses and compromised cellular integrity. We recently discovered that pulmonary Cldn6 expression is significantly inhibited by tobacco smoke [47]. Therefore, the construction of lung-specific transgenic mice that up regulate Cldn6 was undertaken in an effort to counteract Cldn6 targeting by smoke exposure. Intriguingly, Cldn6 mRNA and protein were also significantly diminished in our Cldn6 overexpressing transgenic mice exposed to SHS (Figure 1). It remains probable that additional barrier constituents are differentially impacted by SHS in the lungs of Cldn6 TG mice.

Our research using bronchoalveolar lavage fluid (BALF) demonstrated that inflammatory characteristics are differentially observed in Cldn6 TG vs. control mice. Elevated protein abundance in BALF suggested increased vascular permeability, a finding that coincided with previous research showing cigarette smoke induces BALF protein augmentation [48]. Our discovery that the induction of total cell and PMN diapedesis were decreased in SHS-exposed Cldn6 TG mice reinforced prior research that demonstrated increased PMN admission into the airways and bronchial tissue of smokers diagnosed with COPD [49]. Pro-inflammatory mediators that mechanistically control pulmonary inflammation were also differentially expressed in the lungs of animals exposed to SHS. We assessed the pro-inflammatory effectors TNF-α and IL-1β and discovered that IL-1β was decreased in Cldn6 TG mice exposed to SHS. Known for perpetuating inflammatory axes, TNF-α and IL-1β induce the release of numerous inflammatory cytokines and enhance leukocyte adhesion during chemotactic transmigration. TNF-α can also induce the elaboration of diverse inflammatory and cytotoxic mediators including IL-1β, IL-6, platelet activating factor (PAF), and reactive oxygen species [40]; however such induction is not always correlative. Despite clear implication in the smoke-induced inflammasome, poor correlation between TNF-α and IL-1β was revealed in the current investigation. It is likely that similar patterns of expression and secretion existed between TNF-α and IL-1β at various points of exposure; however, our single assessment at the conclusion of 60 days of exposure did not reveal them. It is also possible that at this snap shot conducted after the 60th day of exposure, TNF-α and IL-1β were differentially impacting aspects of neutrophil chemotaxis and the secretion of other chemokines. Regardless, as we have seen in our smoke exposed mice, TNF-α and IL-1β have been detected in lung cells, BALF, and sputum from COPD patients [50]. Importantly, the detection of these cytokines in BALF or tissues would have been insufficient because both apical and basal elaboration is possible. Therefore, our data intimate that message and protein expression profiles orchestrate wide reaching effects. It is clear that the synthesis and secretion of these and other inflammatory molecules in the context of Cldn6 may specialize the inflammation programs observed in lungs exposed to SHS.

In summary, this research provides a glimpse of the potential impact of Cldn6 in inflammation that follows SHS exposure. It is well understood that smoking is harmful to health; however, debate continues regarding the link between SHS exposure and disease progression. Because Cldn6, even in the TG animal, is inhibited by SHS, much more research is needed that exhaustively considers other tight junctional components and how they participate during inflammation and cancer. Such research may reveal that other molecular constituents in the Cldn6 TG animal are necessary for the anti-inflammatory profiles we have observed. To foreshadow such ongoing endeavors, we have just recently conducted preliminary research that revealed *increased* expression of key tight junctional proteins including occludin [51], ZO-1 [52], tricellulin [53] and JAM-a [14] in the lungs of Cldn6 TG mice exposed to SHS compared to smoked control mice. In a similar fashion, such differential gene expression in Cldn6 TG mice might also explain why the magnitude of Cldn6 inhibition in control mice following SHS exposure was not proportional to what was observed in the exposed TG mice. Additional research should be undertaken that seeks to discern mechanisms that cause smoke-induced inhibition of Cldn6 in over-expressing mice yet leave expression unaffected in smoked controls. It remains notable however that compensatory junctional components may be up-regulated by Cldn6 TG mice during the abrogation of SHS-induced inflammation that may culminate in remodeling or cancer progression.

## 5. Conclusions

This work demonstrates that secondhand smoke (SHS) inhibits Cldn6 expression in lung-specific conditional transgenic mice designed to increase its expression. SHS-induced augmentation of total BALF protein, cells and secreted pro-inflammatory cytokines were all diminished in Cldn6 TG mice exposed to SHS compared to SHS-exposed controls. Intriguing information is provided that suggests roles for Cldn6 in mouse lungs exposed to tobacco smoke. However, further research is needed to fully elucidate discrete roles for this tight junctional protein and other family members in hopes of discovering therapeutically beneficial targets in exposed pulmonary epithelial barriers.
